# Hydrodissection for pericardial adhesion in percutaneous epicardial ventricular tachycardia ablation

**DOI:** 10.1002/joa3.13159

**Published:** 2024-10-10

**Authors:** Yoshimi Onishi, Taku Asano, Shuhei Arai, Yuya Nakamura, Toshiro Shinke

**Affiliations:** ^1^ Division of Cardiology, Department of Medicine Showa University School of Medicine Shinagawa‐ku Tokyo Japan

**Keywords:** catheter ablation, epicardial ventricular tachycardia, hydrodissection, pericardial adhesion, post‐cardiac surgery

## Abstract

Epicardial ablation in patients with pericardial adhesions is challenging. This case is the first report of successful epicardial ventricular tachycardia ablation by combining hydrodissection with the previously reported blunt dissection techniques for pericardial adhesions. This approach demonstrates a promising technique for managing similar cases where traditional methods may fail, providing a safer and more effective solution for epicardial ablation in patients with pericardial adhesions.

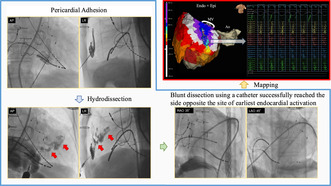

Pericardial adhesions pose significant challenges to epicardial ventricular tachycardia (VT) catheter ablation (CA). Some studies have shown that cautious blunt dissection using guide wires (GW), the curve of the deflected catheter, and steerable sheath can be safely performed in patients with pericardial adhesions.^1^ However, this method has also been associated with a high failure rate^2^ and has not been widely adopted. When catheter‐based pericardial dissection is ineffective, surgical pericardiotomy can be a successful alternative,^3^ though it is more invasive and not commonly performed.

Hydrodissection using pressurized saline is a widely used technique for separating tissue adhesions. This report describes a first case of successful epicardial VT ablation by combining hydrodissection with blunt dissection using catheters through a percutaneous approach.

This 69‐year‐old male underwent mechanical mitral valve replacement for infective endocarditis 6 years ago. Two years after the surgery, he developed congestive heart failure with an ejection fraction of 20% and left bundle branch block, for which a CRT‐D was implanted. Despite this, his cardiac function did not improve, and he developed renal impairment requiring dialysis 3 years later. During dialysis, he experienced VT with a heart rate of 136 bpm and was hospitalized. The VT exhibited a QRS duration of 270 ms and a QS wave in lead I, suggesting an epicardial origin from the left ventricular posterolateral wall. The patient was treated with Pimobendan, Bisoprolol Fumarate for heart failure, and Amiodarone for VT. However, these medications were insufficient to control the VT, leading to the decision to perform catheter ablation.

Given the patient's history, the presence of significant pericardial adhesions was suspected. Therefore, endocardial mapping was initially performed. The VT was reproducibly induced by programmed stimulation and showed progressive fusion on entrainment pacing at different rates, indicating a reentry mechanism. No diastolic potentials were observed endocardially during VT, and activation mapping showed a centrifugal pattern from the left ventricular posterolateral wall, suggesting that the critical isthmus was either epicardial or intramural. Entrainment at the earliest endocardial activation site showed constant fusion, and the post‐pacing interval was 105 ms longer than the tachycardia cycle length. Radiofrequency ablation (40 W for 60 s) at this site did not terminate the VT.

Endocardial mapping indicated that the ablation target was the left ventricular posterolateral wall. Due to the liver's position, accessing the inferior wall was challenging, making only anterior wall access feasible. Using a subxiphoid approach with a 16 G, 90 mm Tuohy needle, pericardial access was attempted. Upon reaching the pericardium, small amounts of contrast medium were injected to confirm minor entry into the pericardial space immediately after the needle pierced the pericardium (Video 1).

**VIDEO 1:** Fluoroscopic video showing initial contrast injection after pericardial access with the Tuohy needle. The contrast medium confirms minor entry into the pericardial space, indicating the needle has pierced the pericardium. All abbreviations are as in Figure 1.

A 0.035‐inch GW was inserted but could only advance 3 cm within the pericardial cavity, indicating significant adhesions. Blunt dissection with the GW was unsuccessful, as evidenced by a lack of contrast spread (Figure 1).

**FIGURE 1:**
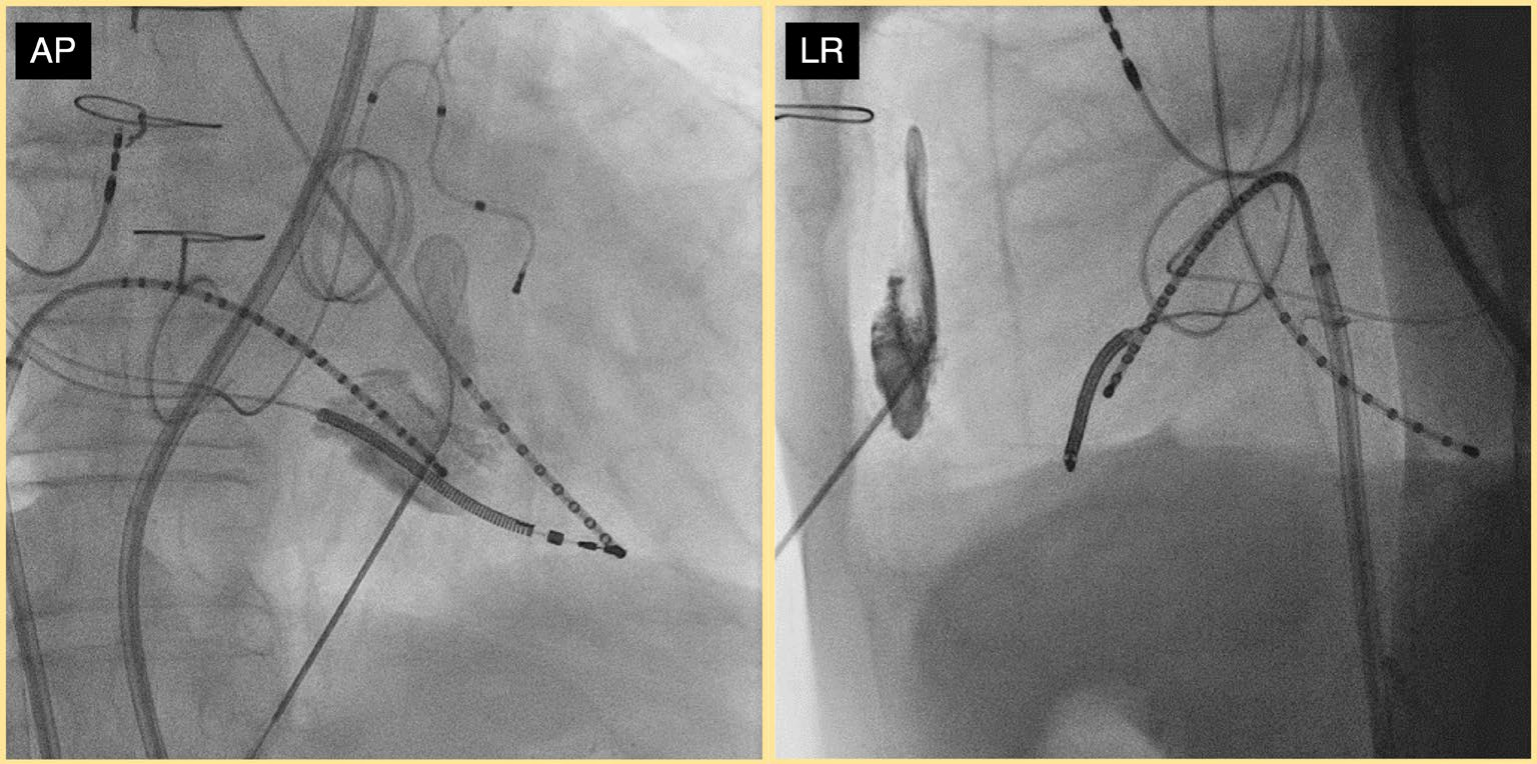
Fluoroscopic image showing the lack of contrast spread after attempted blunt dissection with the guide wire. This indicates significant pericardial adhesions preventing the guide wire from advancing. AP, Anterior–posterior view; LR, left–right view.

With the GW in place, the Tuohy needle was removed, and a 16G, 70 mm indwelling needle cannula was advanced over the GW. In total, 20 mL of 38°C saline with 25% contrast was injected over 5 s, resulting in partial pericardial separation (Video 2).

**VIDEO 2:** Fluoroscopic video showing the hydrodissection with the injection of 20 mL of 38°C warm saline containing 25% contrast medium. The video illustrates partial pericardial separation and widespread contrast distribution. All abbreviations are as in Figure 2.

With further advancement of the GW, an Agilis NxT (Abbott, St. Paul, MN) was inserted. Two additional hydrodissections were performed through the Agilis NxT using similar volumes and injection rates, which facilitated further blunt dissection. Following the direction of contrast spread, blunt dissection was carefully performed using the TactiCath SE (Abbott, Abbott Park, IL) and the flexible 6 Fr decapolar electrode catheter EP Star Snake TS (Japan Lifeline, Tokyo), successfully reaching the posterolateral wall opposite the earliest endocardial activation site (Figure 2).

**FIGURE 2:**
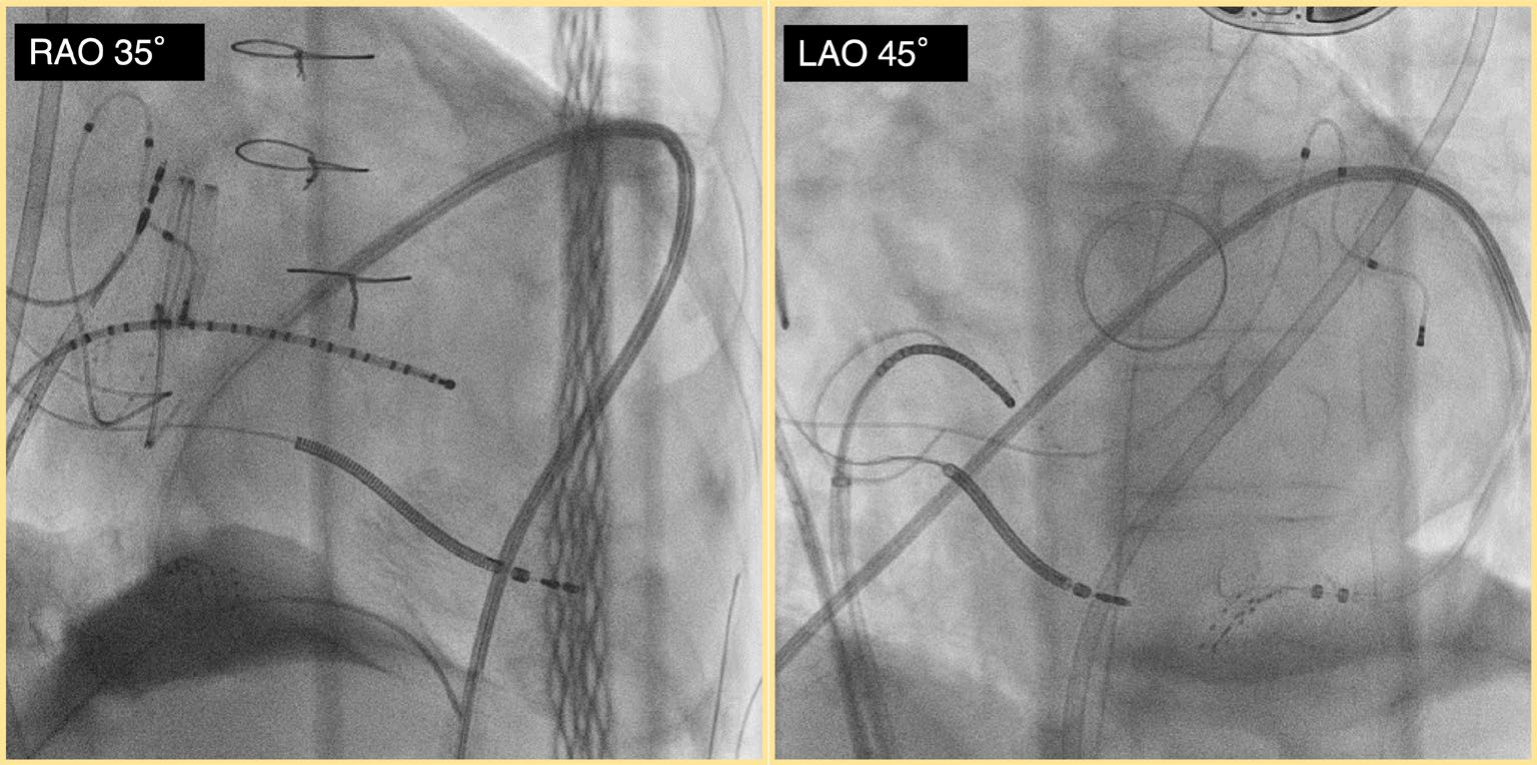
Fluoroscopic image after successful dissection of the pericardial adhesions. After hydrodissection, dissection of the adhesions was performed using a catheter, and finally, the HD grid was inserted for mapping. RAO, right anterior oblique view; LAO, left anterior oblique view.

In this location, diastolic potentials covering the entire VT diastolic phase were observed during VT (Figure 3). The activation map using the Advisor HD Grid (Abbott, St Paul, MN) confirmed that all potentials fulfilling the entire tachycardia cycle were confined to the epicardial side. Entrainment pacing at the slow conduction area shows concealed entrainment (Figure 4). Radiofrequency ablation at this site terminated the VT (Video 3), and no VT was inducible thereafter. The patient remained free of VT recurrence for 2 years post‐ablation. No cardiac injury or significant bleeding occurred during or after the procedure, and the postoperative pericardial drain output was minimal, supporting the safety of the procedure.

**FIGURE 3:**
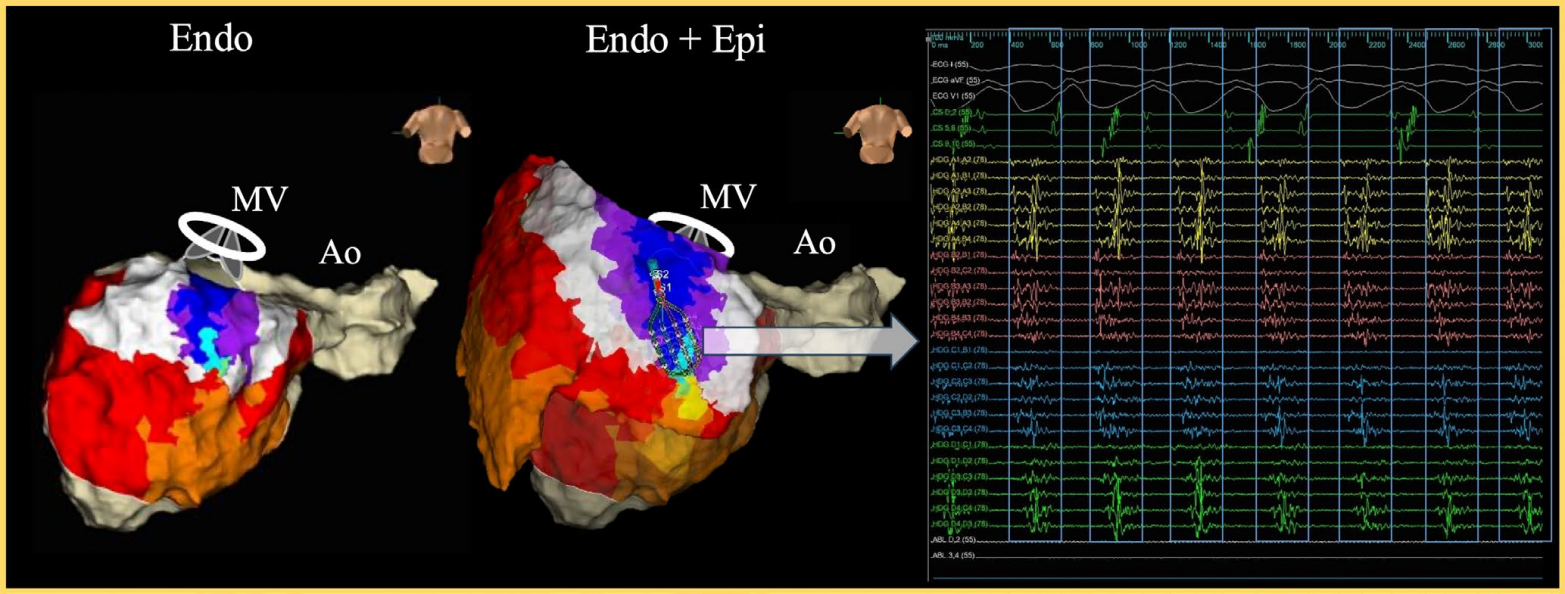
Electrogram showing long diastolic potentials covering the entire VT diastolic phase observed during VT. These potentials indicate the critical isthmus involved in the central isthmus of the reentry circuit. Due to the presence of a mechanical MV in this patient, the endocardial geometry of the left ventricular basal area is limited. The expected position of the mitral valve is indicated schematically in the figure to compensate for this limitation. Ao, aorta; Endo, endocardium; Epi, epicardium; MV, mitral valves; VT, ventricular tachycardia.

**FIGURE 4:**
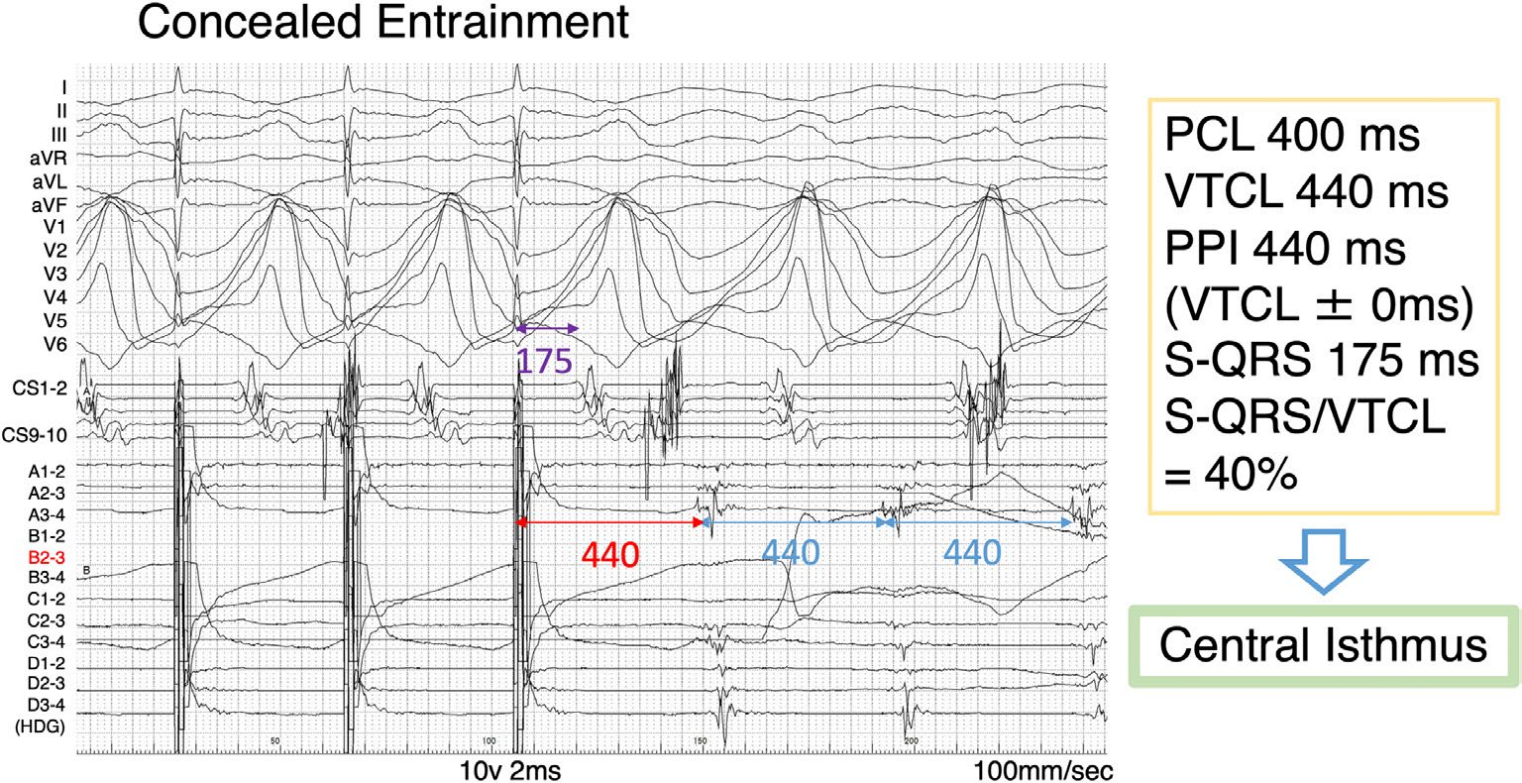
Electrogram showing concealed entrainment with an S‐QRS/VTCL ratio of 40% observed at the slow conduction area. This finding confirms the site of slow conduction within the reentry circuit (central isthmus). CS, coronary sinus; HDG, HD grid; PCL, pacing cycle length; PPI, post‐pacing interval; S‐QRS, stimulus‐QRS time; VTCL, ventricular tachycardia cycle length.

**VIDEO 3:** This video of EnSite shows the termination of VT during ablation at the identified site of slow conduction. All abbreviations are as in Figures 2 and 3.

Mejia et al. reported that hydrodissection can be performed rapidly and safely to separate adhesions between the posterior sternum and the pericardium, as well as between the pericardium and the heart during reoperative cardiac surgery.^4^

Blunt dissection with GW, the curve of the deflected catheter and steerable sheath is often considered effective for percutaneous pericardial adhesion dissection. However, initially, it was unsuccessful in this case. By injecting saline, we increased the pressure within the enclosed pericardial space, promoting separation of the visceral and parietal pericardium. This approach is presumed to be safer than catheter‐based blunt dissection due to the controlled pressure application within the space. While other fluids or CO_2_ gas could potentially be effective, using contrast‐enhanced saline allowed for clear visualization of the dissection progress.

The optimal temperature of saline used in hydrodissection lacks clear evidence. Yokoyama et al. reported that cold saline infusion into the coronary veins temporarily suppressed ventricular premature contractions during CA.^5^ Therefore, we chose warm saline to avoid potential suppression of VT induction and mapping accuracy.

In this case, pericardial access was achieved through the anterior approach, despite the posterior wall being the ablation target. Choosing an access point closer to the predicted ablation target based on the ECG and endocardial mapping data could minimize the dissection area.

Hydrodissection for pericardial adhesion in epicardial VT ablation presents a promising strategy, particularly for patients with post‐surgical adhesions. This technique should be considered before opting for more invasive surgical pericardiotomy or abandoning treatment. While hydrodissection seems safe, the associated blunt dissection with catheters carries a risk of cardiac injury. Therefore, it is crucial to have surgical pericardiotomy readily available as a precaution. Further studies will determine if this approach can become the first choice.

## CONFLICT OF INTEREST STATEMENT

The authors declare no conflict of interest for this article.

## PATIENT CONSENT STATEMENT

The patient has provided consent for publication.

## Data Availability

The data supporting the findings of this study are available from the corresponding author upon reasonable request.

## APPENDIX A

The videos of fluoroscopy before hydrodissection (Video 1), during hydrodissection (Video 2), and the EnSite monitor during ablation (Video 3) are available.
